# Quantitative texture features as objective metrics of enhancement heterogeneity in hypertrophic cardiomyopathy

**DOI:** 10.1186/1532-429X-16-S1-P351

**Published:** 2014-01-16

**Authors:** Rebecca E Thornhill, Myra Cocker, Girish Dwivedi, Carole Dennie, Lyanne Fuller, Alexander Dick, Terrence Ruddy, Elena Pena

**Affiliations:** 1 Medical Imaging, The Ottawa Hospital, Ottawa, Ontario, Canada; 2 Radiology, University of Ottawa, Ottawa, Ontario, Canada; 3 Cardiology, University of Ottawa Heart Institute, Ottawa, Ontario, Canada

## Background

Hypertrophic cardiomyopathy (HCM) results in myocardial disarray, hypertrophy and fibrosis. Late gadolinium enhanced MRI (LGE) can assess the presence and extent of fibrosis, which is associated with the development of arrhythmias and sudden cardiac death. However, enhancement may not always be present or only sparsely distributed. Thus, one of the challenges is how best to describe heterogeneous LGE patterns in an objective fashion that informs clinical decision making. Quantitative texture features may provide clinicians with an objective means of describing the heterogeneity of LGE patterns in HCM. We hypothesized that hypertrophied segments would exhibit greater grey-level heterogeneity than both (a) non-hypertrophied segments in HCM patients, and (b) healthy volunteers.

## Methods

We prospectively recruited 12 HCM patients and 4 healthy volunteers. Functional (bSSFP cine) and LGE (phase-sensitive inversion recovery spoiled GRE, 10-15 min post injection of 0.2 mmol/kg Gd-DTPA) images were acquired in short-axis orientation (SAO), as well as in one 4-chamber slice. We measured the maximum thickness on the end-diastolic cine frame in the 17 segments (AHA model). Segments measuring > 15 mm on SSFP images were considered hypertrophic (H+). Segments were categorized as fibrotic (F+) if > 20% of pixels were enhanced (> 5 SD nulled myocardium). The extent of myocardial fibrosis on LGE imaging and textural features (run-length non-uniformity, RLNU, and grey-level non-uniformity, GLNU [Galloway 1975]) were assessed for each segment. Differences in RLNU and GLNU among segment groups (H+/F+, H+/F-, H-/F+, H-/F-, and healthy) were assessed by Kruskal-Wallis tests.

## Results

**Figure 1:**
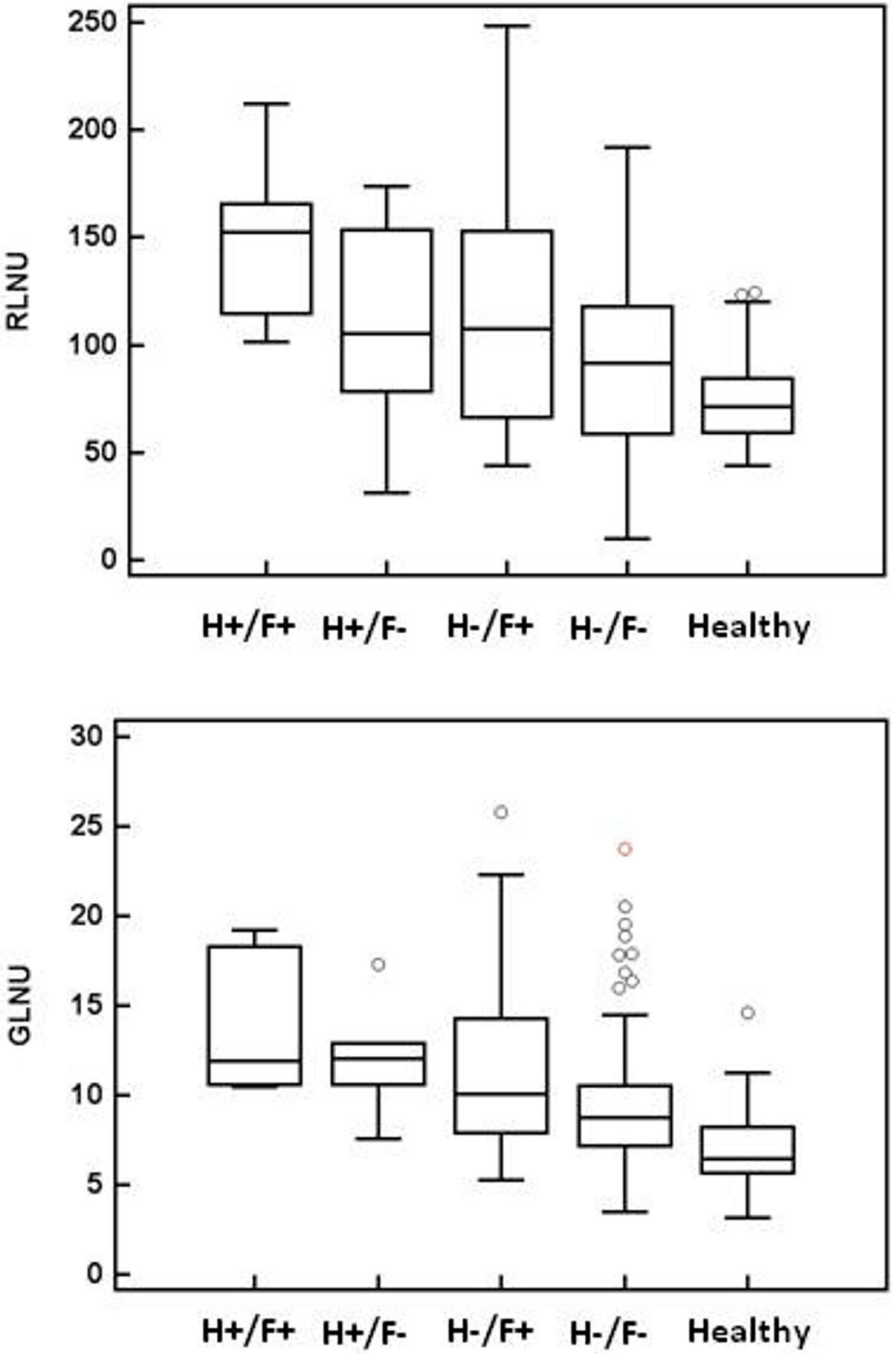
**Box and whisker plots indicating median and interquartile ranges for run-length and grey-level non-uniformity features**: Hypertrophic/Fibrotic (H+/F+), Hypertrophic/Non-Fibrotic (H+/F-), Non-hypertrophic/Fibrotic (H-/F+), Non-hypertrophic/Non-Fibrotic (H-/F-), and Healthy segments.

Of 192 segments we found; 7 H+/F+, 9 H+/F-, 29 H-/F+, and 147 H-/F-. Median +/-interquartile ranges for RLNU and GLNU for each HCM group, as well as for the 64 segments obtained from healthy volunteers are depicted in Figure 1 (P < 0.0001, for RLNU and GLNU). Post-hoc analysis revealed that RLNU and GLNU were significantly greater in H+ than H- segments (P = 0.006 and P = 0.0002, respectively). Both RLNU and GLNU in H-/F- HCM segments were greater than in healthy volunteers (P = 0.009 and P < 0.0001, respectively).

## Conclusions

Quantitative textural features related to LGE heterogeneity appear elevated in patients with HCM, even in non-hypertrophic segments. In addition, significant statistical differences were found in the textural features between non-hypertrophic, non-fibrotic segments of HCM patients and healthy volunteers. Thus, RLNU and GLNU show potential for markers of incipient cardiomyopathic changes among HCM patients and may provide helpful tools for differentiating diverse phenotypic expressions of the disease from healthy patients, pending further validation.

## Funding

Nothing to disclose.

